# Lack of utility of risk score and gynecological examination for screening for sexually transmitted infections in sexually active adolescents

**DOI:** 10.1186/1741-7015-7-8

**Published:** 2009-03-11

**Authors:** Eleuse MB Guimarães, Mark DC Guimarães, Maria Aparecida S Vieira, Nádia M Bontempo, Mirian SS Seixas, Mônica SD Garcia, Lyana ES Daud, Rejane LM Côrtes, Maria de Fátima C Alves

**Affiliations:** 1 Division of Adolescent Medicine, Department of Pediatrics, Faculty of Medicine, Federal University of Goiás, Goiânia, Goiás, Brazil; 2 Department of Preventive and Social Medicine, Faculty of Medicine, Federal University of Minas Gerais, Belo Horizonte, Minas Gerais, Brazil; 3 University Hospital, Federal University of Goiás, Goiânia, Goiás, Brazil; 4 Family Health Program, Goiânia, Goiás, Brazil; 5 Department of Gynecology and Obstetrics, Federal University of Goiás, Brazil; 6 Department of Microbiology, Immunology, Parasitology and Pathology, Institute of Tropical Pathology and Public Health, Federal University of Goiás, Goiânia, Goiás, Brazil

## Abstract

**Background:**

Sexually transmitted infections constitute the main health risk among adolescents. In developing countries the diagnosis and treatment of cervical infections is based on the syndromic approach. In this study we estimated the prevalence of *Chlamydia trachomatis *and *Neisseria gonorrhoeae *among female adolescents from a Health Sector of the city of Goiânia, Brazil, and validated cervicitis diagnosis using World Health Organization/Ministry of Health risk score and gynecological examination.

**Methods:**

A cross-sectional community-based sample of 914 15- to 19-year-old female teenagers was randomly selected and referred to the local Family Health Program. Of these, 472 (51.6%) were sexually active and gynecological examinations were carried out for 427. Endocervical samples were collected to perform the polymerase chain reaction for *C. trachomatis *and *N. gonorrhoeae*. Performance of risk score, the presence of mucopurulent discharge, friability, ectopia and pain during cervical maneuver were compared with the presence of *C. trachomatis *or *N. gonorrhoeae *or both.

**Results:**

The prevalence of *C. trachomatis *and *N. gonorrhoeae *was 14.5% and 2.1%, respectively. The risk score had a specificity of 31.9% (95% confidence interval, 21.2 to 44.2) and a positive predictive value of 20.8% (95% confidence interval, 13.5 to 29.7). Friability was the component of the gynecological examination that presented the best performance with a sensitivity of 43.5%, specificity of 81.0%, and 30.6% of positive predictive value.

**Conclusion:**

The prevalence of infection by *C. trachomatis *and *N. gonorrhoeae *was high among these sexually active adolescents. The syndromic approach is clearly inadequate for screening and treating these infections in this population. Therefore, the implantation of other strategies to control these infections among adolescents is urgently required.

## Background

Sexually transmitted infections (STI) constitute the main health risk among sexually active adolescents [1], as confirmed by data from developed countries where statistical systems are well-organized. In the United States and European countries the infection rates of *Chlamydia trachomatis, Neisseria gonorrhoeae *and human papillomavirus are highest among adolescents and young adults up to 24 years old [2,3]. In addition, it is well recognized that STI may increase the likelihood of human immunodeficiency virus (HIV) transmission as much as 20-fold [4]. In developing countries, including Brazil, although available data are scarce, these trends were confirmed [5,6].

Sexually active adolescents should be adequately targeted for prevention and treatment of STI by early screening strategies [7]. However, in many developing countries, especially those in Africa [8], where the acquired immune deficiency syndrome (AIDS) epidemic has reached catastrophic proportions, lack of adequate diagnostic resources jeopardize this strategy. The World Health Organization (WHO) has proposed a syndromic approach for screening and treating STI, which uses algorithms favoring specific symptoms such as urethral discharge, the presence of ulcers and abnormal vaginal discharge [9]. The first two have proven to be efficacious for treatment purposes [10-12]. However, the algorithm abnormal vaginal discharge, which has been validated in several studies, is currently criticized, especially because the most prevalent STI are frequently asymptomatic [13,14]. In addition, this algorithm has never been validated in Brazil in an exclusively adolescent population; such validation is also rare worldwide [15,16]. The aim of the present study was to validate the diagnosis of cervicitis due to *C. trachomatis and N. gonorrhoeae *using the WHO/Ministry of Health risk score and gynecological examination in a population-based sample of female adolescents in Brazil.

## Methods

### Setting and study design

The study was a population-based cross-sectional study of sexually active female adolescents resident in the Northeastern Health Sector of Goiânia, a city with 1,129,274 inhabitants in the central region of Brazil. The overall population of this sector was 11,389 inhabitants, with an estimated total of 4091 15 to 19-year-old female adolescents attending a Family Health Program.

The sample size was estimated as 588 female sexually active adolescents, 15 to 19 years old, considering a 1.5% precision, minimum expected STI prevalence of 4%, and an anticipated refusal rate of 10%. Households with potential participants were randomly selected from census tract information provided by the local health department using a systematic sampling scheme.

All female adolescents in each selected household were invited by letter to come to the health center nearest to their residence. At the health center, they were informed about the study, and were initially interviewed by a physician trained in adolescent medicine or a trained nurse, using a questionnaire containing sociodemographic and general health-related questions, including the onset of sexual activity. Those sexually active were invited for further procedures, including a gynecological examination, while those who were sexually inactive were counseled concerning general health issues or referred for a medical appointment and/or vaccination whenever necessary. Adolescents who had used antimicrobial drugs within the previous 15 days as well as pregnant women were excluded from enrollment. Those eligible adolescents were requested to sign a written informed consent and the study was approved by the Ethical Review Board from the University Hospital, Federal University of Goiás after proper authorization by Goiânia City Health Department. Interviews were carried out in private rooms and confidentiality was assured.

### Gynecological examination

Sexually active adolescents were referred to a physician for a gynecological examination. They also answered a more specific questionnaire concerning sexual practices, reproductive life and gynecological symptoms. The risk score was then determined based on the following items: partner with urethral discharge, 2 points; age less than 20 years-old, 1 point; more than one partner in the last three months, 1 point; new partner in the last three months, 1 point; no fixed partner, 1 point. The risk score was considered positive when equal to or greater than 2 points [11].

An examination of the abdomen, external and internal genitalia, followed by a speculum examination was performed on each subject. After cleaning the ectocervix, material was collected by introducing a swab into the endocervix and then rotating for 5 seconds before careful removal. Samples were immediately placed in a polymerase chain reaction (PCR) transportation tube for PCR testing for *C. trachomatis *and *N. gonorrhoeae*. The gynecological examination data assessed for validation of a diagnosis of cervicitis were those proposed by the Ministry of Health: mucopurulent endocervical discharge, cervical friability and pain upon bimanual maneuver of the cervix, plus the presence of ectopia.

### Laboratory method

The samples for PCR testing were transported to the Cellular Immunology Laboratory of the Institute of Tropical Pathology and Public Health of the Federal University of Goiás, Brazil. PCR for *C. trachomatis *and *N. gonorrhoeae *were carried out by the CT/NG Amplicor kit (Roche Molecular Systems, Branchburg, NJ, USA) according to the manufacturer's instructions using standard procedures. Internal controls were used in all amplifications.

### Data management and analysis

The prevalence of *C. trachomatis *and *N. gonorrhoeae *infections and their respective 95% confidence intervals (95% CI) were estimated as the number positive in each PCR test divided by the total number of adolescents tested. Sensitivity, specificity, positive predictive value (PPV), negative predictive value, with their respective 95% CI were estimated to determine the ability of risk score and gynecological examination components to differentiate between those infected with either *C. trachomatis or N. gonorrhoeae *and those uninfected cases. PCR results for both infections were the gold standard for comparisons. Differences in proportion were assessed using Fisher's exact test. Data were analyzed using Epi Info, version 6.0 (CDC, Atlanta, GA, USA).

## Results

In order to achieve the necessary sample size, 1539 female adolescents belonging to the randomly selected households were invited for a medical visit at their nearest health unit. From August 2002 to September 2003, 914 (60.0%) adolescents aged 15 to 19 years attended the health units and were eligible for the study. The main reason for not responding was a change of address (*N *= 335), while 166 were not eligible due to age criteria, 89 were pregnant and 35 had other reasons.

Among the 914 adolescents who fulfilled the inclusion criteria and gave informed consent 472 (51.6%) were sexually active and 427 (90.5%) of these had a gynecological examination performed, including the collection of material for the diagnosis of a chlamydial and gonococcal infection. Only four teenagers refused to participate in the study and 41 were excluded because they were menstruating or had used oral medications or vaginal topical antibiotics in the last 15 days.

Sociodemographic data indicated a uniform distribution across ages (mean age = 17.2 ± 1.3 years), and revealed that an important proportion of these teenagers were already married or living with partners (32.1%). Most of them had between 5 and 8 years of schooling, had poorly educated mothers and low family income (Table 1)

**Table 1:** Sociodemographic characteristics of 427 sexually active adolescents resident in the Northeastern Health Sector of Goiânia, Goiás, Brazil, in 2003.

**Variables**	***N***	**%**
*Age (years old)*		
15	54	12.6
16	86	20.1
17	93	21.8
18	108	25.4
19	86	20.1
*Marital status*		
Single	290	67.9
Married/cohabiting	137	32.1
*Schooling (years)*		
<5	19	4.5
5 to 8	224	52.5
>8	183	42.8
not available	1	0.2
*Mother's schooling (years)*		
None	37	8.7
<5	183	42.8
5 to 8	145	34.0
>8	39	9.1
not available	23	5.4
*Family income (minimum wages)*		
<2 MWs	200	46.8
2 to 4 MWs	155	36.3
>4 MWs	48	11.3
not available	24	5.6

MW = 153 US$

### The WHO/Ministry of Health risk score and gynecological examination data

The prevalence of infection with *C. trachomatis *was expressively high (14.5%; 95% CI 11.4 to 18.3), while the prevalence of *N. gonorrhoeae *was markedly lower (2.1%; 95% CI 1.0 to 4.1). Only two adolescents presented with a simultaneous *C. trachomatis *and *N. gonorroeae *infection.

Overall, 69 (16.2%) adolescents were positive for *C. trachomatis *and/or *N. gonorrhoeae *while the risk score (≥ 2 points) was positive in 106 (24.8%; 95% CI 20.8 to 29.2) subjects. The majority (64.1%) of adolescents with a positive risk score had only 2 points. The frequency of a positive risk score, pain upon bimanual maneuver, ectopia, friability or mucopurulent discharge was higher in those subjects positive for an STI (Table 2). However, only pain, friability and mucopurulent discharge were statistically different between groups (*P *< .05).

**Table 2:** Frequency of risk score and gynecological examination data among adolescents infected with *Chlamydia trachomatis *and/or *Neisseria gonorrhoeae*.

**Variables**	***C. trachomatis *and/or *N. gonorrhoeae *positive *N *= 69** ***N *(%)**	**No STI** ***N *= 358** ***N *(%)**	**Fisher's exact test** ***P *value**
*Risk score*^a^			
Positive	22 (31.9)	84 (23.5)	.170
*Pain upon bimanual maneuver*			
*Yes*	25 (36.2)	71 (19.8)	.0044^b^
*Ectopia*			
Yes	21 (30.4)	89 (24.9)	.367
*Friability*			
Yes	30 (43.5)	68 (19.0)	<.0001^c^
*Mucopurulent discharge*			
Yes	11 (15.9)	28 (7.8)	.040^d^

^a^Positive risk score: ≥ 2 points.

^b^Odds ratio = 2.297, 95% confidence interval = 1.318, 4.003.

^c ^Odds ratio = 3.281, 95% confidence interval = 1.903, 5.655.

^d ^Odds ratio = 2.235, 95% confidence interval = 1.054, 4.738.

The performance of the risk score and gynecological examination data regarding cervical infection diagnosis is described in Table 3. Sensitivity of the WHO risk score algorithm was 31.9%, that is, only 22 out of 69 women with a cervical infection were correctly identified. The specificity was 76.5% and the PPV was 20.8%, that is, 20.8% of those identified by the WHO risk score actually had a cervical infection. Friability was the component of the gynecological examination that presented the best performance with a sensitivity of 43.5%, specificity of 81.0% and 30.6% of PPV. Finally, after testing for combinations of clinical signs and risk score, there was no indication of improvement in sensitivity for detecting STI among these female adolescents.

**Table 3:** Sensitivity, Specificity, Positive Predictive Value and Negative Predictive Value of the Risk Score and Gynecological Examination Data for Diagnosing Cervicitis by *Chlamydia Trachomatis *and/or *Neisseria Gonorrhoeae*.

**Variables**	**Sensitivity** **% (95% CI^a^)**	**Specificity** **% (95% CI)**	**PPV**^b^ **% (95% CI)**	**NPV^c^** **% (95% CI)**
*Risk score*	31.9 (21.2–44.2)	76.5 (71.7–80.8)	20.8 (13.5–29.7)	85.4 (81.0–89.0)
*Pain upon bimanual maneuver*	36.2 (25.6–48.7)	80.2 (75.6–84.2)	26.0 (17.6–36.0)	86.7 (82.6–90.2)
*Ectopia*	30.4 (19.9–42.8)	75.1 (70.3–79.5)	19.1 (12.2–27.7)	84.9 (80.5–88.6)
*Friability*	43.5 (31.5–56.0)	81.0 (76.6–84.9)	30.6 (21.7–40.7)	88.2 (84.2–91.5)
*Mucopurulent discharge*	15.9 (8.2–26.7)	92.2 (88.9–94.8)	28.2 (15.0–44.9)	85.1 (81.1–88.4)

CI: confidence interval

PPV: positive predictive value

NPV: negative predictive value

## Discussion

This study was a community-based representative sample of the female adolescent population, aged 15 to 19 years old, residents of one of the most socially deprived regions of the city of Goiânia. The prevalence of infection, particularly with *C. trachomatis*, was very high.

It is well known that adolescents present the highest prevalence levels of these infections in most countries [17,18]. Moreover, the fact that this population is one of low family income and lower level of education further increases the risk of STI [19,20].

In Brazil, recent data indicate that the fastest growing rate of HIV infection is among adolescents aged 15 to 19 years-old [21]. Thus, the control of STI is imperative, not only because of their intrinsic complications, especially in relation to chlamydia and gonococcus infection among female adolescents, but also due to their facilitating action in relation to HIV infection [22-25].

STI screening programs in many developed and developing countries, including Brazil, are not fully implemented. In the United States, for example, the Centers for Disease Control recommend annual screening of chlamydia infection among sexually active female adolescents and young adults up to 25 years of age, but this program is not widely followed [26,27].

Despite the national and international recognition of the Brazilian National AIDS Program, Brazil is lagging behind other countries in regard to the detection and treatment of other STI, including secondary prevention. Only recently, a more clear priority has been given to implementing public health policies for the control of STI, including chlamydia and gonococcus infections. The current choice for STI treatment adopted by the Ministry of Health is the use of a syndromic approach for diagnosing and treating cervicitis, despite it being known that these infections are asymptomatic in up to 80% of cases [28].

Our results indicate that the use of the risk score for cervicitis screening and diagnosis among female adolescents is not adequate due to the large proportion of asymptomatic infections, most of which are positive for chlamydia. The low sensitivity and low PPV of the risk score should be of major public health concern. In addition, the risk score does not appear to improve the management of cervical infections, and should not be used as a screening tool or a diagnostic test among asymptomatic or poorly symptomatic women [29,30]. Components of the gynecological examination assessed for validation of a cervicitis diagnosis proposed by the Ministry of Health also had a poor performance.

## Conclusion

The prevalence of infection by *C. trachomatis *was alarmingly high. The risk score proposed by the Ministry of Health and the findings of gynecological examinations are not adequate for the diagnosis and treatment of cervicitis due to chlamydia and gonococcus infection in adolescents. Therefore, other strategies to control these infections among adolescents are urgently required. Among such strategies, emphasis must include investment in primary prevention through sexual education, which should include the family, school, health services and sexual partners. The 'reinvention' of sexual education is also a fundamental requirement, since the currently used methods, while achieving increased awareness among young people, especially regarding HIV infection, has not achieved the desirable and essential level of a change in behavior [31].

As far as secondary prevention is concerned, the development of rapid and less costly tests for chlamydia and gonococcus diagnosis in resource-poor public health services are urgently needed. Studies regarding the prevalence of chlamydia and gonococcus infection should be encouraged in other regions of Brazil so that improved clinical-epidemiological diagnosis of this highly relevant public health problem occurs at a national level, similar to developed countries.

## Abbreviations

AIDS: acquired immune deficiency syndrome; CI: confidence interval; HIV: human immunodeficiency virus; PCR: polymerase chain reaction; PPV: positive predictive value; STI: sexually transmitted infections; WHO: World Health Organization

## Competing interests

The authors declare that they have no competing interests.

## Authors' contributions

EMBG and MFCA were responsible for the study design, data collection and data analyses, and drafted the manuscript. MDCG contributed to the study design and was responsible for the epidemiological aspects, and helped to draft the manuscript. MASV collaborated in the elaboration of the instruments for collecting sociodemographic and sexual behavior data and participated in the fieldwork. NMB collaborated in the design of the study regarding aspects related to the syndromic approach. MSSS and MSDG participated in data collection and the realization of gynecological examinations. LESD and RLMC realized the laboratory examinations and collaborated in the discussion of the results.

## Pre-publication history

The pre-publication history for this paper can be accessed here:


